# A Semantic Segment Encoder (SSE): Improving human face inversion quality through minimized learning space

**DOI:** 10.1371/journal.pone.0295316

**Published:** 2023-12-05

**Authors:** Byungseok Kang, Youngjae Jo

**Affiliations:** R&D Center, Dob Studio, Mapo-gu, Seoul, Republic of Korea; Shandong Normal University, CHINA

## Abstract

Recently, Generative Adversarial Networks (GAN) has been greatly developed and widely used in image synthesis. A Style-Based Generator Architecture for Generative Adversarial Networks (StyleGAN) which is the foremost, continues to develop human face inversion domain. StyleGAN uses insufficient vector space to express more than one million pixels. It is difficult to apply in real business due to distortion-edit tradeoff problem in latent space. To overcome this, we propose a novel semantic segment encoder (SSE) with improved face inversion quality by narrowing the size of restoration latent space. Encoder’s learning area is minimized to logical semantic-segment units that can be recognized by humans. The proposed encoder does not affect other segments because only one segment is edited at a time. To verify the face inversion quality, we compared with the latest encoders both Pixel2style2Pixel and RestyleEncoder. Experimental result shows that the proposed encoder improved distortion quality around 20% while maintain editing performance.

## 1. Introduction

Generative Adversarial Networks (GANs) [1] is applied not only to image creation and restoration, but also to non-image fields such as voice generation or editing, new drug development, and prediction. In 2018, NVIDA developers announced StyleGAN [2, 3] which generates high-performance real-world images. It is actively used in GAN Inversion research domain. Despite the excellent performance of StyleGAN, detailed inversion (accuracy improvement) of real images remains a difficult task. Inversion for high frequencies in a narrow area such as eyes or teeth shows an unnatural.

StyleGAN inversion has been studied into an encoder-based and optimization-based method. Encoder-based method has high inversion speed but lacks quality (accuracy). It learns how to invert images directly into latent space, and after learning, it can solve the given task through short-time reasoning. Conversely, the optimization-based method has excellent quality but slow inversion speed. Optimization-based methods mainly start from a specific latent code and optimize to move to a place where similar images can be produced. This optimization process takes a lot of time because it is repeated from 300 to 1000 times.

In the study of latent space of StyleGAN, *W* space is insufficient to express all images, and it is common to expand and invert to *W*^+^ space. In some cases, a study using a space larger than *W*^+^ by directly injecting features into the generator have been proposed. As the size of the latent space increases, the distortion performance improves, but editing becomes more difficult. As a compromise solution for this distortion-edit tradeoff, e4e [4] is to use latent codes that are as close to the *W* space as possible. This study slightly reduces distortion quality but could have more advantages for editing. In [5], they maintain editing performance while improving quality of distortion by optimizing local segment.

In this paper we introduce a Semantic Segment Encoder (SSE) which meets the high quality of distortion and editing performance. Proposed encoder has three advantages: 1) By reducing the learning range through segmentation, high restoration quality and wide range of editing are possible. 2) Expands the selection range of localization editing by selectively inverting and editing segments. 3) Supports high quality 2D/3D application in any field of academy and industry.

## 2. Related work

This chapter mainly introduces GAN Inversion-related studies. We explain distortion-edit tradeoff, an important issue in GAN Inversion. We also describe the advantages and disadvantages of the recently introduced StyleGAN3 [6].

### 2.1 GAN inversion

GAN Inversion has been studied steadily for a long time, and it has received more attention as StyleGAN. Inversion is mainly divided into three categories. First, the ‘optimization’ takes a long time but has higher reconstruction performance. StyleGAN2 [3] starts from a random latent code and optimizes it with a latent code that generates more similar images. As mentioned above, this optimization-based method has high reconstruction accuracy, but since it takes a lot of time for one image, it is often inefficient for videos that need to process many inputs.

The second, after learning a specific network with the ‘encoder’, it has a reconstruction performance that is inferior to the optimization method instead of having a very short inversion time. pSp [2] extracts the features of the image in more detail through the residual structure and infers the latent code into *W+* space through each map2style layer. ReStyle [7] infers a more detailed latent code than before by restoring the difference from a specific face to a target face. BaseLine [8] has high performance while lightening the model by reducing the number of heads and a structure more suitable for feature map extraction in the architecture of pSp. This encoder-based method can invert an image into a latent vector in a runtime time of around 1 second, so it is suitable for videos that need to process multiple images at once.

Finally, the ‘hybrid’ approach is a combination of encoder-based and optimization-based methods. [9, 10] use an encoder to generate an initial latent code, and the next optimization program uses it as a starting point to further refine the latent code. In addition, PTI [11] achieved high reconstruction accuracy by obtaining an approximate latent code of a real image and then tuning the generator. It is faster than the optimization-based method and has good reconstruction performance. Segment divides the segment to optimize the latent code, and at the same time find and synthesize a segment with a smooth boundary while improving the segment. As a method of improving performance by reducing the area to be inverted, the optimization method is basically used, and images are created with very high reconstruction performance. The method presented in this paper is inspired by this study, but it is faster because segment is redefined to fit the encoder-based method. In [12], they introduce a high-fidelity face swapping that faithfully preserves the desired subtle geometry and texture details. Compared to the latest techniques, they showed excellent performance in preserving texture and shape details, as well as working with high resolution images. In [13], they present a novel GAN inversion framework that enables attribute editing with image-specific details well-preserved. They propose an adaptive distortion alignment (ADA) module with a self-supervised training scheme. They showed significant improvement both inversion and editing quality.

### 2.2 Editing and distortion-edit tradeoff

Inversion of StyleGAN mainly consists of *W* space or *W+* space. The *W* space mainly composed of 512 dimensions has excellent editing performance but has low reconstruction performance due to insufficient space to express an image of 1024x1024 resolution. Face Identity [14] proposed a method of learning by separating the identity and attribute of an image in the *W* space and exchanging only the attribute while maintaining the identity.

The *W+* space has higher reconstruction performance with more dimensions, but with more risk in editing. This inversely proportional relationship between reconstruction performance and editing performance is called distortion-edit tradeoff, most studies mainly adopt this space. According to the GAN Inversion Survey [15], recently proposed methods use the *W+* space. ISE [16] proposes a method that greatly improves editing performance with little loss of reconstruction performance through PCA-based $P_{N}^{+}$ space and regularization in this space. Based on the architecture of pSp, e4e infers the latent Code as a *W+* space close to *W* space, rather than a general *W+* space, and presents a compromise solution for distortion-edit tradeoff. Additionally, there are studies that perform inversion in feature map units instead of latent Code. StyleMapGAN [17] induces natural blending between images by up-sampling *W* defined as R^64x8x8^ and injecting the extracted feature map into the StyleGAN Generator. However, the feature map unit method does not support general editing mode.

### 2.3 StyleGAN3

StyleGAN has gained great popularity because it can generate high-definition images while having disentangle characteristics due to the mapping network and the structure of inserting style vectors into each stage that gradually grows. Recently, StyleGAN3 proposed by Karras et al. pointed out that there is a texture sticking problem in StyleGAN2, and the StyleGAN3 Model that solved this problem was proposed. The ‘Texture Sticking’ problem occurs when Generator uses unintended location information. Very detailed textures such as hair, wrinkles, and eyebrows stick to the screen and do not move naturally with the rest of the object, resulting in unnatural images. In StyleGAN3, to solve this problem, starting with a thorough analysis of the structure of the StyleGAN2 generator, all layers of the generator have equivariant properties.

In the GAN inversion process, which mainly used the StyleGAN2 generator, a texture sticking problem occurred, and it caused fatally unnatural images to be generated in continuous images. To solve this problem, simply using the StyleGAN3 generator results in a loss of reconstruction accuracy according to [18], resulting in a more unnatural image than before. For example, although the actual image is a sideways gaze, the generated image may generate a frontal image, or there may be problems such as having many teeth or wrong positions unlike the actual image. If the inversion performance is forcibly raised to solve this problem, the editing performance may decrease rapidly due to the distortion-edit tradeoff.

### 2.4 Summary

GAN inversion has been studied mainly by dividing it into an encoder-based method that is fast but lacks performance and an optimization-based method that has excellent performance but is slow. The Encoder-based method learns how to invert images directly into latent space, and after learning, it can solve the given task with a short amount of reasoning. Optimization-based methods mainly start from a specific latent code and optimize to move to a latent code that can produce an output image. This optimization process takes a lot of time because it goes from 300 times to 1000 times at the shortest. A hybrid method utilizing the advantages of the two methods has recently attracted a lot of attention.

In previous studies on inversion and StyleGAN’s *W* is insufficient to express all images, and it has become a common case to invert by extending it to *W*+ space. In some cases, they use a space larger than *W*+, directly injects features into the generator to increase inversion performance. What can be learned from these studies is that the larger the size of the space, the better the reconstruction performance, but the more difficult the editing. Recently, many studies proposed a way to overcome the distortion-editing tradeoff and use more resources but still have several quality issues. In this paper, we have demonstrated that proposed SSE improved reconstruction performance while maintaining editing performance.

## 3. Semantic Segment Encoder (SSE)

The proposed method applies a mask of a specific part to the output of existing encoder-based models. Only the corresponding area becomes the output of the model, and the remaining areas use the original image. This reduces the area where the Encoder will extract information in the learning stage, enabling more detailed inversion for that range. In this chapter, we discuss the process of dividing segments in order, the learning method, and the loss function.

### 3.1 Face parsing

**Fig 1:**
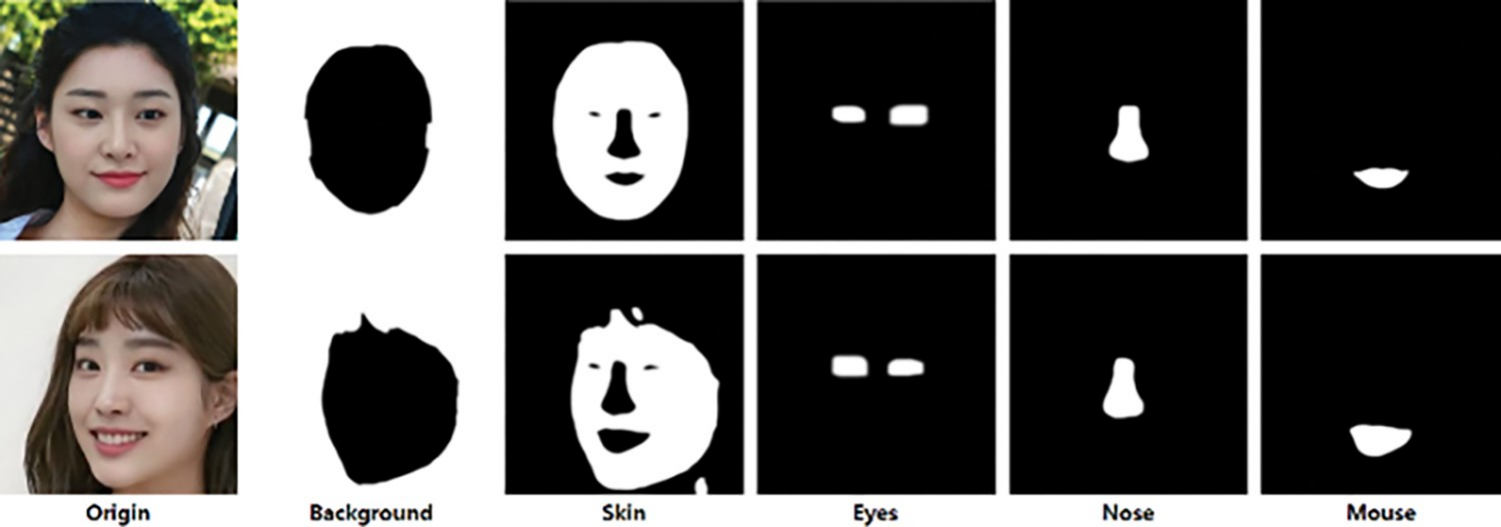
Result of mask generation through face-parsing. Reprinted from [Lee] under a CC BY license, with permission from [PLOS ONE], original copyright [2023].

Our goal is to properly divide the segment so that the encoder learns and inverts as much information as possible. To maximize this information, it is divided into the most parts, and it must be divided with a criterion that cannot change for most images. Therefore, Real Image is segmented as much as possible while having meaning, but eyes are an exception as they have pairs. In this paper, a total of *k* = 5 segments is used, which are divided into skin, eyes, nose, mouth, and background according to the entangled area of StyleGAN that can be analyzed in StyleSpace [19]. In this paper, face-parsing is used to segment this area, and one face is divided into four areas as shown in Fig 1. For natural combining, the parsing domain is expanded using the Dilate operation. In particular, the eye has a narrow parsing area, so it executes 15 times stronger expansion operations. A mask *M*^*i*^ corresponding to each segment is created. The input image is expressed as (1).


$$P(x)=\sum_{i=1}^{k}M^{i}$$ (1)

### 3.2 Training

We defined the model *E_base*, which will be the basis for all learning. In this paper, pSp, e4e, restyle is adopted as the architecture of *E_base*, and StyleGAN3 is adopted as the decoder. Since it is not possible to express Real Image with high quality in StyleGAN3 only with the information that *E_base* can acquire from Real Image, additional learning is conducted. Additional learning was performed 60,000–100,000 times per model. The parameters and process of additional learning proceed the same as *E_base*, and the image generated by calculating the mask on the resulting image is different. The segment model $E_{seg}^{i}$ created by *E_base* additional learning receives a Real Image as an input and creates an image and exchanges the Segment Mask M^i^ corresponding to each model with the Real Image. The output of each model for training is as follows.


$${\hat{y}}_{seg}^{i}=G(E_{seg}^{i}(x))*M^{i}+x*(1-M^{i})$$ (2)

In Eq (2), *x* is the input image, *G* is the generator, $E_{seg}^{i}$ encode the *i*^th^ segment, and ${\hat{y}}_{seg}^{i}$ is the output of the *i*^th^ segment. The final output that can be generated by combining the outputs of each model after learning all *k* models in total is as follows.


$$\hat{y}=\sum_{i=1}^{k}G(E_{seg}^{i})*M^{i}$$ (3)

### 3.3 Loss function

Most studies use three loss functions: pSp, e4e, and restyle. Basically, L2 loss to reduce reconstruction loss in pixel units, LPIPS Loss to reduce perceptual reconstruction loss, and e4e use w_regularization loss to generate latent code close to *W* space. As mentioned in e4e, the adversarial loss using Discriminator is not used because it greatly degrades the quality empirically.

Preserving the key information of the face is a very important task in GAN inversion. Therefore, the given face is encoded using ArcFace [20] and the cosine similarity between the faces is compared. Unlike the commonly used method, by using all the results according to the resolution mentioned in Baseline [8], the loss that all five feature maps including the output layer are used is calculated. To more accurately calculate the id loss (identification information) between the reconstructed image and the input image according to the resolution size, five feature maps are used. The total loss function is defined as Eq (4). Encoder loss is calculated by L2 + LPIPS. The id loss is obtained as the difference between the reconstructed image and the input image. Algorithm 1 shows the process of segment learning.


$$L_{\;}=\;\lambda_{encoder}L_{encoder}\;+\lambda_{id}L_{id}$$ (4)

**Algorithm 1**. pseudo-code of Segment Learning.

**Input**: the training image set X, the traininge mask set M, pre-trained model *G*, encoder E, maximum number of iterations *T*, restoration output y

**Output**: encoder E^+^

Let E^0^← E

for t = 1 to *T* do

*x*~*X*

*m*~*M*

y=G(E(x))*m+x*(1−m) //calculate restoration output

Calculate *L* as in Eq (4) //calculate total loss

*E*^(*t*+1)^ is updated with *δ*ℒ/*δ*E^(t)^.

*E*^+^←*E*^*t*+1^

## 4. Experimental results

To verify the performance of the proposed method, we compare it qualitatively and quantitatively with recently proposed encoder such as pSp, e4e, and ReStyle. Encoder Backbone uses SE-ResNet50 Backbone, and StyleGAN3-config-R model is used as generator. For segment, face parser is used, and among the loss functions, AlexFace is used for Alex and Id Loss for LPIPS Loss. Encoder learning is also conducted with FFHQ Dataset generator [21]. The evaluation uses the CelebA-HQ Dataset [22] and selects 100 random images. All experiments were conducted on a single NVIDIA Tesla P100 16GB GPU.

### 4.1 Qualitative comparison

**Fig 2:**
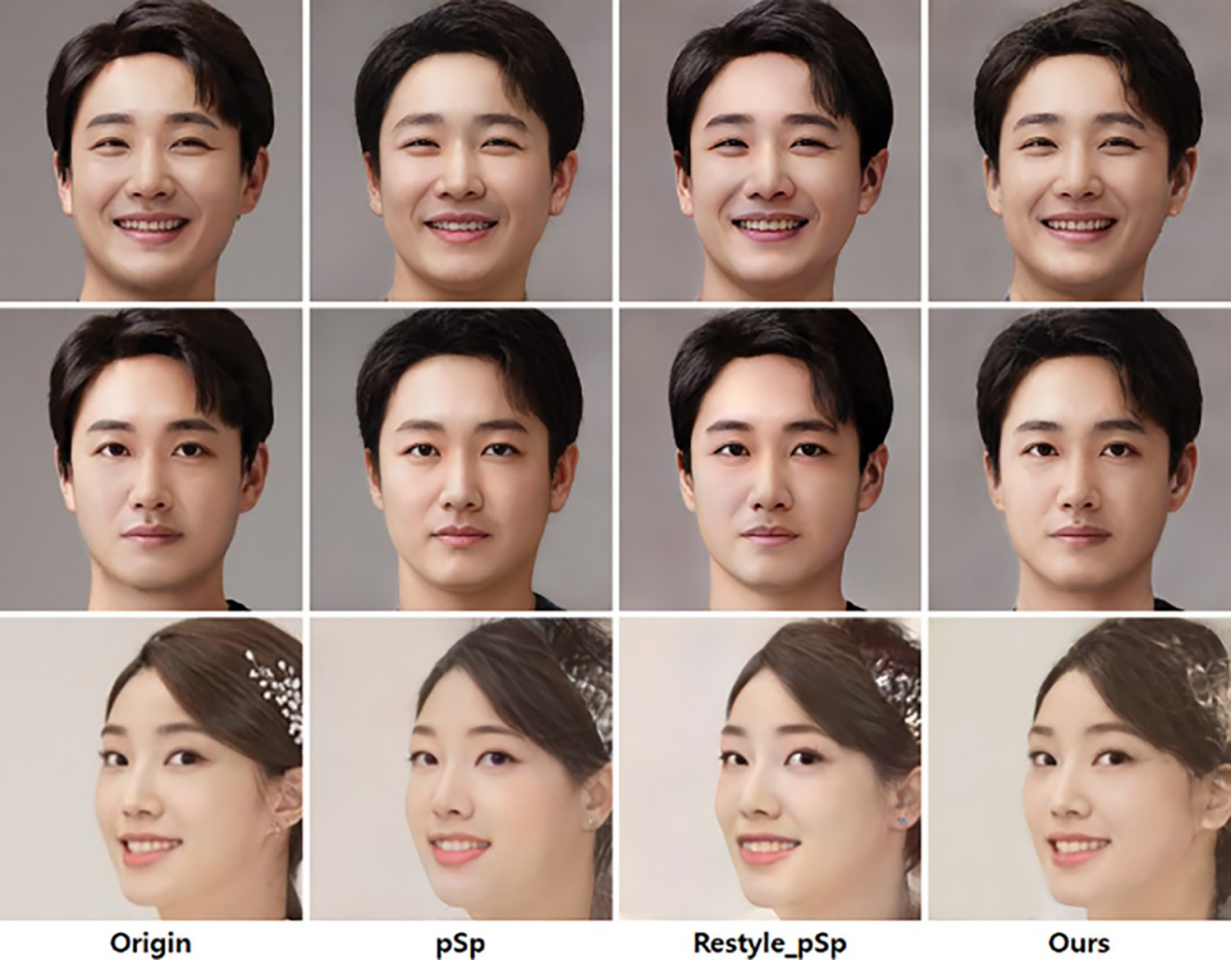
Input (origin) and output images of three encoders. Reprinted from [Lee and Oh] under a CC BY license, with permission from [PLOS ONE], original copyright [2023].

Qualitative evaluation is a subjective human evaluation. In the field of generative AI, the uncanny valley that people visually feel is an important evaluation factor. Fig 2 shows the qualitative evaluation results. Although e4e has a similar structure to pSp, it gives up reconstruction performance and improves editing performance, so only pSp is used in the evaluation of GAN Inversion, and e4e is not compared. Compared to pSp and Restyle, our method inverts more accurate color and overall impression.

As can be seen in the 2^nd^ and 3^rd^ rows of Fig 2, detailed information such as the position of pupils and teeth in the face area is composed more accurately and in detail than the Restyle method. In the first row of the left figure in Fig 2, the eyes of the input image and the image of SSE are directed to the front, while the eyes of Restyle are directed to the right. The gaze of a person in a video is very important information, and even a slight pixel difference gives a completely different gaze. Proposed method can create an image with a gaze without distortion with high reconstruction performance in a detailed area.

### 4.2 Quantitative comparison

We compare our method quantitatively with other recent studies. In this evaluation, pSp and Restyle networks learned with FFHQ Dataset were used. For the above examples, Structural Similarity Index Measure (SSIM), Learned Perceptual Image Patch Similarity (LPIPS) distance, and Peak Signal-to-noise ratio (PSNR) scores are calculated and reported respectively. PSNR has been widely used in various digital image measurements and has been considered tested and valid. The smaller the loss (the better the image quality), the higher the value. SSIM is designed based on luminance, contrast, and structure. LPIPS essentially computes the similarity between the activations of two image patches for some pre-defined network. This measure has been shown to match human perception well. The ID is the rate at which the person can be recognized. The measurement result reports the average time of 50 iterations of inference on a single machine.

In Table 1, SSE shows higher performance in SSIM, PSNR, and ID indicators. This result proves that proposed method has higher reconstruction performance than Restyle. In LPIPS, pSp was measured high, but there was no significant difference from SSE. However, since the proposed idea divides the face into segments and restores it, the reconstruction time (runtime) is longer than other models. According to the results reported by the charm [18], the inversion performance of StyleGAN3 is about 10% inferior to that of StyleGAN2 inversion, but overcoming this and achieving a high index can be seen as a very positive result. Because StyleGAN3 uses multiple models for one inference, it has the disadvantage of using more cost compared to other encoder-based methods.

**Table 1:** Results of measurements on 100 random copies of the Celeba-HQ Dataset.

	SSIM	PSNR	LPIPS	ID	Runtime(sec.)
pSp	0.47	62.99	0.15	0.81	0.16
Restyle_pSp	0.50	63.92	0.12	0.89	0.74
Ours(SSE)	0.54	64.58	0.13	0.93	2.24

### 4.3 Mask boundary artifact testing

**Fig 3:**
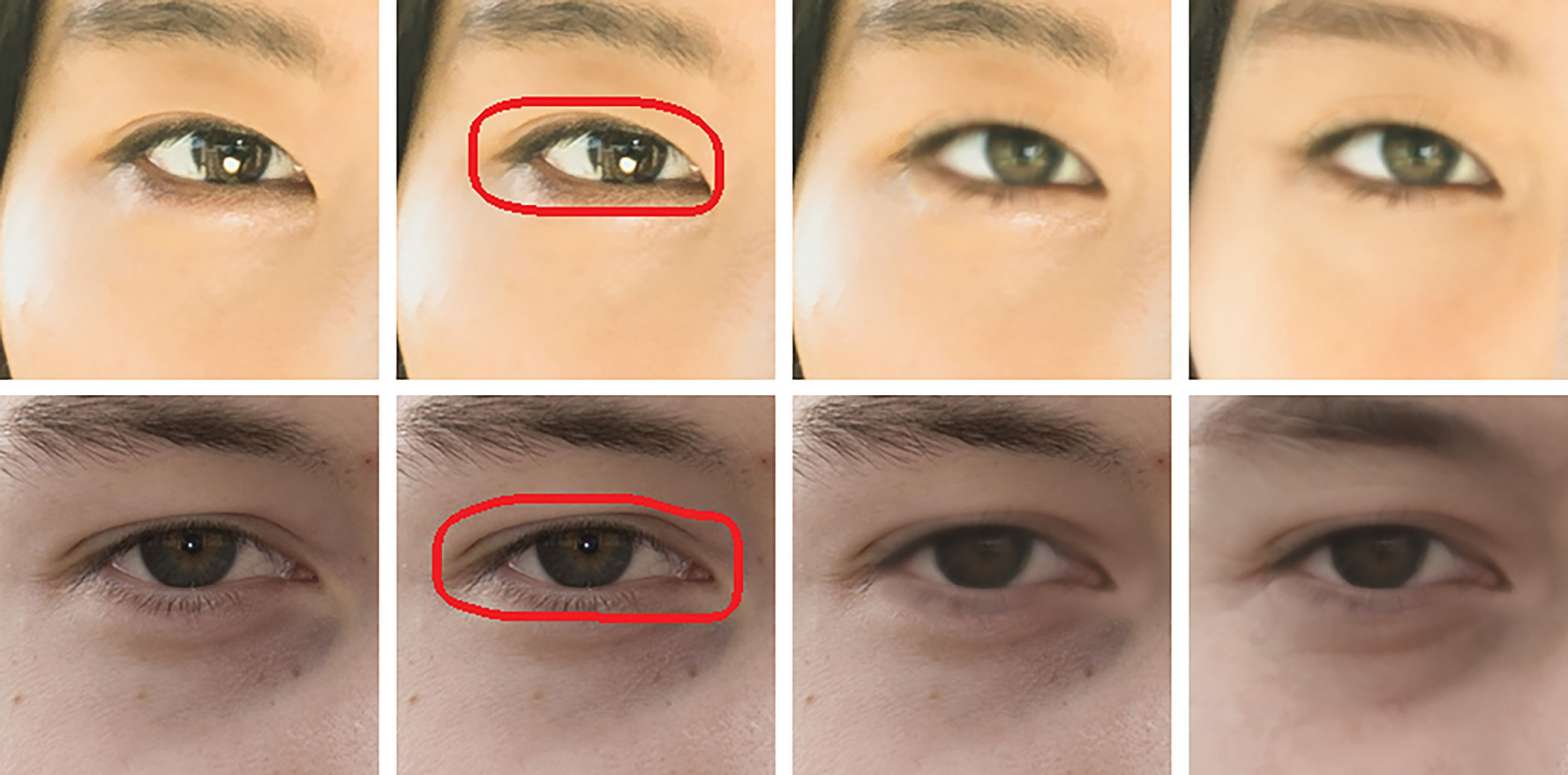
Column 1: Actual image, Column 2: Boundary of the synthetic area on the actual image, Column 3: Combining real image and synthesized image, Column 4: Combining the synthesized images. Reprinted from [Oh] under a CC BY license, with permission from [PLOS ONE], original copyright [2023].

As can be seen from the previous results, the output generated by the model sufficiently trained by our method does not generate visually identifiable artifacts when the mask area is exchanged with a real image. For more detailed analysis, check if the two prerequisites mentioned in Chapter 2 are satisfied. 1) Whether Synthesis Image and Real Image are combined without creating a boundary. 2) Whether the Synthesis Images are combined without creating a boundary. The qualitative evaluation results are shown in Fig 3, and the quantitative evaluation results are shown in Table 2, respectively. For quantitative evaluation, the range of the mask boundary area is obtained for 100 random images in the Celeba HQ dataset, and the error rate calculated by the difference within boundary area. In Table 2, the smaller the boundary difference is the more natural the generated face is. The previous algorithm generated a mask boundary of approximately 0.02, and the SSE generated around 0.01. In other words, SSE showed a performance improvement of 2 times compared to the existing algorithms.

**Table 2:** Distance between the real image and the mask boundary area of the composite image.

	pSp(SG3)	pSp(SG2)	Ours(SSE)
Boundary Difference	0.0216	0.0203	0.0127

The first prerequisite is confirmed by combining synthesis image and real image. From a qualitative point of view, as shown in column 3 of Fig 3, the synthesized image does not produce a visible border. SSE produced significantly better results than Restyle-pSp(SG3) and Restyle-pSp(SG2) in quantitative measurement. Segment learning is specialized for mask boundaries and induces natural combining. The second precondition is confirmed by combining synthesis images. As you can see in column 4, it does not create a visible boundary line. Each segment model learned to invert close to the real image does not require special artifacts even when different outputs are combined. This proves that a sufficiently natural image can be generated with a simple BitMask operation without any special image synthesis logic and reconstructs the input image with a very high similarity.

## 5. Conclusion

This paper proposed a segment-encoder SSE that improves the reconstruction performance of the face generation encoder. This makes it possible to encode more detailed contents by reducing the range of information included in latent code. The model learned in this way can reverse the image within the domain without additional learning, and can express details of the local area, such as the position of the pupil or teeth, which existing encoders could not solve, in much more detail. Through experiments and sequential image synthesis, the proposed method improved restoration performance around 20% compared to the existing method.

There are still have several research challenges. Inversion of each segment is simply a pixel unit operation. If the outline or color of the face is heavily edited, a boundary line or unnatural image is created. In addition, the editing performance between the models corresponding to each segment is different, so there may be a gap between the user’s intention and the segment. To solve these problems, we have a plan to add a render network [23, 24].

## References

[1] Creswell A., White T., Dumoulin V., Arulkumaran K., Sengupta B., & Bharath A. A. (2018). Generative adversarial networks: An overview. IEEE signal processing magazine, 35(1), 53–65.

[2] Richardson E., Alaluf Y., Patashnik O., Nitzan Y., Azar Y., Shapiro S., et al. (2021). Encoding in style: a stylegan encoder for image-to-image translation. In Proceedings of the IEEE/CVF conference on computer vision and pattern recognition (pp. 2287–2296).

[3] Karras T., Laine S., Aittala M., Hellsten J., Lehtinen J., & Aila T. (2020). Analyzing and improving the image quality of stylegan. In Proceedings of the IEEE/CVF conference on computer vision and pattern recognition (pp. 8110–8119).

[4] Tov O., Alaluf Y., Nitzan Y., Patashnik O., & Cohen-Or D. (2021). Designing an encoder for stylegan image manipulation. ACM Transactions on Graphics (TOG), 40(4), 1–14.

[5] Šubrtová A., Futschik D., Čech J., Lukáč M., Shechtman E., & Sýkora D. (2022, October). ChunkyGAN: Real Image Inversion via Segments. In Computer Vision–ECCV 2022: 17th European Conference, Tel Aviv, Israel, October 23–27, 2022, Proceedings, Part XXIII (pp. 189–204). Cham: Springer Nature Switzerland.

[6] Karras T., Aittala M., Laine S., Härkönen E., Hellsten J., Lehtinen J., et al. (2021). Alias-free generative adversarial networks. Advances in Neural Information Processing Systems, 34, 852–863.

[7] Alaluf Y., Patashnik O., & Cohen-Or D. (2021). Restyle: A residual-based stylegan encoder via iterative refinement. In Proceedings of the IEEE/CVF International Conference on Computer Vision (pp. 6711–6720).

[8] Wei T., Chen D., Zhou W., Liao J., Zhang W., Yuan L., et al. (2021). A simple baseline for stylegan inversion. arXiv preprint arXiv:2104.07661, 9, 10–12.

[9] Abdal R., Qin Y., & Wonka P. (2019). Image2stylegan: How to embed images into the stylegan latent space?. In Proceedings of the IEEE/CVF International Conference on Computer Vision (pp. 4432–4441).

[10] Abdal R., Qin Y., & Wonka P. (2020). Image2stylegan++: How to edit the embedded images?. In Proceedings of the IEEE/CVF conference on computer vision and pattern recognition (pp. 8296–8305).

[11] Roich D., Mokady R., Bermano A. H., & Cohen-Or D. (2022). Pivotal tuning for latent-based editing of real images. ACM Transactions on Graphics (TOG), 42(1), 1–13.

[12] Liu Z., Li M., Zhang Y., Wang C., Zhang Q., Wang J., et al. (2023). Fine-Grained Face Swapping via Regional GAN Inversion. In Proceedings of the IEEE/CVF Conference on Computer Vision and Pattern Recognition (pp. 8578–8587).

[13] Wang T., Zhang Y., Fan Y., Wang J., & Chen Q. (2022). High-fidelity gan inversion for image attribute editing. In Proceedings of the IEEE/CVF Conference on Computer Vision and Pattern Recognition (pp. 11379–11388).

[14] Nitzan Y., Bermano A., Li Y., & Cohen-Or D. (2020). Face identity disentanglement via latent space mapping. arXiv preprint arXiv:2005.07728.

[15] Xia W., Zhang Y., Yang Y., Xue J. H., Zhou B., & Yang M. H. (2022). Gan inversion: A survey. IEEE Transactions on Pattern Analysis and Machine Intelligence. 10.1109/TPAMI.2022.3181070 37022469

[16] Zhu P., Abdal R., Qin Y., Femiani J., & Wonka P. (2020). Improved stylegan embedding: Where are the good latents?. arXiv preprint arXiv:2012.09036.

[17] Kim H., Choi Y., Kim J., Yoo S., & Uh Y. (2021). Exploiting spatial dimensions of latent in gan for real-time image editing. In Proceedings of the IEEE/CVF Conference on Computer Vision and Pattern Recognition (pp. 852–861).

[18] Alaluf Y., Patashnik O., Wu Z., Zamir A., Shechtman E., Lischinski D., et al. (2023, February). Third time’s the charm? image and video editing with stylegan3. In Computer Vision–ECCV 2022 Workshops: Tel Aviv, Israel, October 23–27, 2022, Proceedings, Part II (pp. 204–220). Cham: Springer Nature Switzerland.

[19] Wu Z., Lischinski D., & Shechtman E. (2021). Stylespace analysis: Disentangled controls for stylegan image generation. In Proceedings of the IEEE/CVF Conference on Computer Vision and Pattern Recognition (pp. 12863–12872).

[20] Deng J., Guo J., Xue N., & Zafeiriou S. (2019). Arcface: Additive angular margin loss for deep face recognition. In Proceedings of the IEEE/CVF conference on computer vision and pattern recognition (pp. 4690–4699).

[21] Nguyen T. H., Van Le T., & Tran A. (2023). Efficient Scale-Invariant Generator with Column-Row Entangled Pixel Synthesis. In Proceedings of the IEEE/CVF Conference on Computer Vision and Pattern Recognition (pp. 22408–22417).

[22] Yang Q., Pu Y., Zhao Z., Xu D., & Li S. (2023). W2GAN: Importance Weight and Wavelet feature guided Image-to-Image translation under limited data. Computers & Graphics. doi: 10.1016/j.cag.2023.08.016

[23] Lazova V., Guzov V., Olszewski K., Tulyakov S., & Pons-Moll G. (2023). Control-nerf: Editable feature volumes for scene rendering and manipulation. In Proceedings of the IEEE/CVF Winter Conference on Applications of Computer Vision (pp. 4340–4350). doi: 10.48550/arXiv.2204.10850

[24] Abbas F., & Babahenini M. C. (2023). Forest fog rendering using generative adversarial networks. The Visual Computer, 39(3), 943–952.

